# Identification and Characterization of Three Spore Wall Proteins of *Enterocytozoon Bieneusi*

**DOI:** 10.3389/fcimb.2022.808986

**Published:** 2022-06-20

**Authors:** Xinan Meng, Haojie Ye, Ziyu Shang, Lianjing Sun, Yaqiong Guo, Na Li, Lihua Xiao, Yaoyu Feng

**Affiliations:** ^1^ Center for Emerging and Zoonotic Diseases, College of Veterinary Medicine, South China Agricultural University, Guangzhou, China; ^2^ Guangdong Laboratory for Lingnan Modern Agriculture, Guangzhou, China

**Keywords:** microsporidia, *E. bieneusi*, spore wall protein, immunofluorescence, detection

## Abstract

*Enterocytozoon bieneusi* is the most common microsporidian pathogen in farm animals and humans. Although several spore wall proteins (SWPs) of other human-pathogenic microsporidia have been identified, SWPs of *E. bieneusi* remain poorly characterized. In the present study, we identified the sequences of three *E. bieneusi* SWPs from whole genome sequence data, expressed them in *Escherichia coli*, generated a monoclonal antibody (mAb) against one of them (EbSWP1), and used the mAb in direct immunofluorescence detection of *E. bieneusi* spores in fecal samples. The amino acid sequence of EbSWP1 shares some identity to EbSWP2 with a BAR2 domain, while the sequence of EbSWP3 contains a MICSWaP domain. No cross-reactivity among the EbSWPs was demonstrated using the polyclonal antibodies generated against them. The mAb against EbSWP1 was shown to react with *E. bieneusi* spores in fecal samples. Using chromotrope 2R staining-based microscopy as the gold standard, the sensitivity and specificity of the direct immunofluorescence for the detection of *E. bieneusi* were 91.4 and 73.7%. Data generated from the study could be useful in the characterization of *E. bieneusi* and immunological detection of the pathogen.

## Introduction

*Enterocytozoon bieneusi* is emerging as an important zoonotic pathogen in recent years (Matos et al., 2012). It colonizes in the intestine of a wide range mammals and birds, causing diarrhea in humans and farm animals. It is responsible for approximately 90% of reported cases of human microsporidiosis (Han and Weiss, 2017). *E. bieneusi* infections are mostly limited to the gastrointestinal tract; in those with immunocompromising conditions it can cause severe or chronic diarrhea, malabsorption and wasting (Han et al., 2021).

*Enterocytozoon bieneusi* belongs to the Enterocytozoonidae family, which contains several genera of microsporidia of mammals, birds, fish and arthropods (Li et al., 2021). The genus *Enterocytozoon* has three established species, with the other ones including *E. hepatopenaei* in shrimps and *E. schreckii* in salmons (Tourtip et al., 2009; Couch et al., 2022). In addition, recent genetic characterizations at multiple loci indicate that the canine-adapted Group 11 genotypes of *E. bieneusi* might represent a third species (Ou et al., 2021). Like other microsporidia, *E. bieneusi* has a hardy environmental stage, the spore. Coiled inside the spore is an organelle unique to microsporidia, the polar tube, which is used in delivering the sporoplasm into the host cells during invasion (Vávra and Larsson, 2014).

The spore wall of microsporidia is composed of the plasma membrane, endospore, and exospore (Han et al., 2020). As an organelle of direct contact with the host and external environment, the spore wall plays an important role in the survival and invasion of the pathogen (Yang et al., 2018). Numerous spore wall proteins (SWPs) have been identified in human-pathogenic *Encephalitozoon* spp., including EcSWP1, EcSWP3/EcEnP2, EcSWP4/EcEnP1, EcCDA, and EcSWP5 from *E. cuniculi* (Katinka et al., 2001; Brosson et al., 2005; Peuvel-Fanget et al., 2006; Xu et al., 2006); EiSWP1, EiSWP2, and EiEnP1 from *E. intestinalis* (Hayman et al., 2001; Corradi et al., 2010; Pombert et al., 2012); and EhSWP1a and EhSWP1b from *E. hellem* (Polonais et al., 2010). Furthermore, the first spore wall protein of *E. hepatopenaei* (EhSWP1) has been characterized recently. It is expressed in both the endospores and exospore (Jaroenlak et al., 2018). The genetic relatedness of *E. hepatopenaei* make it an ideal model in studying the biology of *E. bieneusi* (Ou et al., 2021).

In the present study, we identified three SWPs from the genomic data of *E. bieneusi* (Akiyoshi et al., 2009), expressed them in *Escherichia coli*, and assessed the cross-reactivity among them using the polyclonal antibodies generated. We have further developed a monoclonal antibody (mAb) against one of them, used it in the development of an immunofluorescence assay for the detection of *E. bieneusi* spores in fecal samples, and compared its performance with the conventional modified chromotrope 2R staining.

## Materials and Methods

### Identification and Structural Prediction of EbSWPs

We searched for SWPs of *E. bieneusi* (EbSWPs) in the partial genomic data of *E. bieneusi* (Akiyoshi et al., 2009) at MicrosporidiaDB (https://microsporidiadb.org/micro/app/) using a combination of text search and blastp analysis using the amino acid sequence of EhSWP1 (Jaroenlak et al., 2018). InterPro (https://www.ebi.ac.uk/interpro/) was used to identify the function domains in the amino acid sequences of three EbSWPs and two EhSWPs. MEME Suite (https://meme-suite.org/meme/tools/meme) was used to further identify sequence motifs in these SWPs. The online software NetNGlyc (http://www.cbs.dtu.dk/services/NetNGlyc/) and NetOGlyc (http://www.cbs.dtu.dk/services/NetOGlyc/) were used to predict the N-glycosylation sites and O-glycosylation sites in the EbSWPs, respectively. The isoelectric point and molecular weight of proteins were predicted using ExPASy (http://web.expasy.org/compute_pi/), while signal peptide and transmembrane domains were be identified using SignalP (http://www.cbs.dtu.dk/services/SignalP/) and TMHMM V2.0 (http://www.cbs.dtu.dk/services/TMHMM-2.0/), respectively.

The online server I-Tasser (https://seq2fun.dcmb.med.umich.edu//I-TASSER/) was used to predict the structure of the full length of EbSWPs using the default parameters (Zheng et al., 2021). The model with the highest confidence score (C-score in the range of -5 to 2, with C-score >-1.5 indicating a model of the correct global topology) among all the results was selected for each protein. The I-Tasser models generated were downloaded in the PDB format and modified visually using Chimera 1.16 (https://www.cgl.ucsf.edu/chimera/download.html).

### Molecular Cloning, Expression, and Purification of Recombinant EbSWPs

PCR was used to amplify the full-length EbSWP genes, including EBI_25395 (encoding EbSWP1), EBI_25820 (encoding EbSWP2), and EBI_25393 (encoding EbSWP3). The DNA was extracted from one fecal sample that was positive for *E. bieneusi*. The primers used in the amplification of the EBI_25395 gene included SWP1-F 5’-CGGGATCCTATCAAGAAACAAAAAGATATATTG-3’ (with the *Bam*H I restriction enzyme site underlined) and SWP1-R 5’-TTGTCGACATTAAATTTTTCAAGATGGTTTC-3’ (with the *Sal* I restriction enzyme site underlined). The PCR program included one cycle of 98°C for 30 s; 35 cycles of 98°C 10 s, 55°C for 30 s, and 72°C for 30 s; and one cycle of 72°C for 5 min. The PCR was performed using the Phusion™ High-Fidelity DNA Polymerase (ThermoFisher, Waltham, MA, United States), with the product of the expected size being purified using the MiniBEST DNA Fragment Purification Kit (TaKaRa, Kyoto, Japan). Similarly, the EBI_25820 gene was amplified using primers SWP2-F 5’- CGGGATCCGAAACAGTAACTAATAATAATATATC-3’ (with the *Bam*H I restriction enzyme site underlined) and SWP2-R 5’- TTGTCGACAACAAATATATCTAATTCATCAG-3’ (with the *Sal* I restriction enzyme site underlined), and the EBI_25393 gene amplified using primers SWP3-F 5’-CGGGATCCTTCAATTTTACATTAATAGCGC-3’ (with the *Bam*H I restriction enzyme site underlined) and SWP3-R 5’- TTGTCGACTAAACTGAGTATATTGAAATAAC-3’ (with the *Sal* I restriction enzyme site underlined).

For expressing recombinant SWPs, BL21 (DE3) competent cells of *E. coli* were transformed with the recombinant EbSWP-pCold I vectors with a His-tag incorporated and cultured in LB medium supplemented with 100 µg/mL ampicillin. The expression of EbSWPs was induced by adding 1 mM isopropylthio-β-galactoside (IPTG) to the cultures, which were subsequently maintained at 16°C for specified duration. Recombinant EbSWPs in culture lysates were examined using SDS-PAGE and Western blot analyses with anti-His-tag antibodies (Cell Signaling Technology, Beverly, MA, United States).

For protein purification, cells in the BL21(DE3) cultures were collected by centrifugation and lyzed by ultrasonication on ice at 45% maximum output (setting 6) for 20 min on a cell destructor (Shanghai Jinxin Industrial Co., China), with each cycle consisting of five 2-second pulses separated by 7-second rest. Following sonication, the lysate was centrifuged for 20 min at 10,000 × *g*, and the supernatant generated was filtered through a 0.45 µm polyvinylidene fluoride (PVDF) membrane (Millipore, Billerica, MA, United States). The filtrate was loaded onto a column containing Ni-NTA His-bind resins (General Electric Company, Syracuse, NY, United States). Recombinant EbSWPs were eluted from the resins with imidazole and examined using SDS-PAGE and Western blot analyses.

### Western Blot Analysis

In SDS-PAGE analysis of recombinant EbSWPs, 40 μL protein was added into 10 μL 5×SDS loading buffer and incubated in a water bath at 100°C for 10 min. After polyacrylamide gel electrophoresis of the mixture, the protein in the gel was transferred onto a polyvinylidene fluoride membrane (Millipore). The membrane was blocked with 5% skimmed milk powder for 2 h and reacted with mouse anti-His-tag antibodies (Cell Signaling Technology). Peroxidase-conjugated goat anti-mouse IgG (Beyotime Technology, Shanghai, China) was used as the secondary antibody.

### Polyclonal Antibody Production

The purified recombinant EbSWPs (1 mg/mL) were emulsified with an equal volume of Freund’s complete and incomplete adjuvants (Sigma, St. Louis, MO, United States) to immunize four-week-old female specific pathogen-free rabbits. The immunization was conducted by subcutaneous injection of 0.5 mL emulsified antigen. Five immunizations were conducted with an interval of one week each. Approximately 40 days after the primary immunization, sera were isolated from rabbits. An enzyme-linked immunosorbent assay (ELISA) was used to determine the titer of polyclonal antibodies (pAb) in the serum of immunized rabbits. The specificity of the antibodies generated was assessed using Western blot analysis with recombinant EbSWPs.

### Monoclonal Antibody Production

Three 5-week-old female BALB/c mice were immunized subcutaneously five times at 2-week intervals with 200 μg EbSWP1 emulsified 1:1 in Freund’s complete adjuvant (Sigma) for the primary immunization or Freund’s incomplete adjuvant (Sigma) for subsequent immunizations. The level of antibodies against EbSWP1 was monitored using ELISA. Before the monoclonal antibody (mAb) generation, immunized mice were boosted intraperitoneally with 100 μg EbSWP1. The mice were euthanized 3 days later, and the spleen cells were harvested and fused with SP2/0 cells as described (Gefter et al., 1977). Positive clones of hybridoma cells were identified using ELISA.

The specificity of the mAb generated was assessed using ELISA and Western blot analysis. In ELISA, wells in 96-well plates were coated with the antigen under analysis. The mAb was used as the primary antibody, while the peroxidase-conjugated goat anti-rabbit IgG (Beyotime) as the secondary antibody. Other antigens to be tested included lysates of *Cryptosporidium parvum* oocysts and *E. coli* cells, as well as recombinant EbSWP2 and EbSWP3. Western blot analysis was further conducted on EbSWP1, EbSWP2, and EbSWP3 using mAb against EbSWP1 as the primary antibody and procedures described above.

### ELISA

ELISA was used to identify positive clones of hybridomas and measure the titers of pAb and mAb generated. ELISA plates were coated with 0.4 μg recombinant proteins/well. The plates were incubated at 37°C in a wet box for 2 hours. After washing, 1% BSA-PBS was added to the wells to block nonspecific binding of the ELISA plates for 2 h. For serum titer determination, immune and preimmune sera were serial-diluted prior to the addition of ELISA wells. Horseradish peroxidase-conjugated goat-anti-rabbit IgG or goat-anti-mouse IgG (Beyotime) was used as the secondary antibody in ELISA. The optical density (OD) of the reaction was measured at 450 nm in a microplate reader (BioTek, America). For the identification of the positive hybridoma clones, 100 μL culture was used as the source of the primary antibodies in ELISA. Positive (post-immune serum at 1:1,000 dilution) and negative (pre-immune serum or media from SP2/0 cell cultures) controls were included in each ELISA test. The hybridoma clones were identified as positive when the OD values obtained were at least three times greater than those from negative cultures.

### Direct Immunofluorescence Detection of *E. bieneusi* Spores

The mAb (at the concentration ≥ 2 mg/mL) generated was cross-linked in 0.1 M sodium carbonate buffer (pH 9.8) with fluorescein isothiocyanate isomer I (FITC) (Sigma). For each 1 mL mAb solution, 50 µL FITC dissolved in anhydrous dimethyl sulfoxide at the concentration of 1 mg/mL was added, with constant shaking during the process. The mixture was incubated overnight at 4°C in the dark. Afterwards, 50 mM NH_4_Cl was added to the mixture, which was incubated at 4°C for 2 h. Xylene cyanol FF and glycerol were added to the mixture at the final concentrations of 0.1% and 5%, respectively. The FITC-conjugated mAb was desalted using a desalting spin column (ThermoFisher).

For the detection of *E. bieneusi* spores in fecal samples by direct immunofluorescence microscopy using the FITC-conjugated mAb, 20 μL fecal suspension was smeared evenly on a high-absorption glass slide and air-dried. The slide was flame-fixed and blocked with 1% BSA at 37°C for 30 min. FITC-labeled mAb against EbSWP1 was added to the smears at 1:100 dilution, and incubated at 37°C for 1 h. After washing off unbound antibodies, the slides were mounted with No-Fade Mounting Medium (Booster, Wuhan, China) and examined under a BX53 fluorescence microscope (Olympus, Tokyo, Japan). *Enterocytozoon bieneusi* spores in 48 samples from crab-eating macaque monkeys in Haikou, Hainan, China and 48 samples from calves in Zhaoqing, Guangdong, China were detected using the mAb-based immunofluorescence microscopy. For comparison, these samples were also analyzed by bright-field microscopy after the chromotrope 2R-staining of fecal smears as described (Weber et al., 1992). The sensitivity, specificity, and positive and negative values of the immunofluorescence microscopy using the chromotrope 2R-based microscopy as the gold standard were calculated as per standard formulae. The McNemar’s test implemented in SPSS version 26 (SPSS, Inc., Chicago, IL, USA) was used to assess the agreement between the two techniques, with a *p* value <0.05 being considered significant.

## Results

### SWPs in *E. bieneusi*

The blastp analysis of the genomic data of *E. bieneusi* in MicrosporidiaDB using the EhSWP1 sequence of *E. hepatopenaei* led to the identification of EbSWP1 encoded by the EBI_25395 gene and EbSWP2 encoded by the EBI_25820 gene. The text search of the MicrosporidiaDB database led to the identification of an additional EbSWP: EbSWP3, which is encoded by the EBI_25393 gene. The latter is shortly upstream from EBI_25395 and the annotation of protein in MicrosporidiaDB indicates that it encodes a protein containing a SWP domain (pfam17018).

### Sequence Characteristics and Tertiary Structure of EbSWPs

The full-length open reading frame of the gene for EbSWP1 is 687 bp, encoding a protein of 228 amino acids, with a molecular mass of 26.8 kDa and a theoretical isoelectric point of 7.06. In contrast, the full lengths of the genes for EbSWP2 and EbSWP3 are 744 and 690 bp, encoding proteins of molecular masses of 29.2 and 25.9 kDa and theoretical isoelectric points of 9.46 and 5.15, respectively. The 88th amino acid of EbSWP1 may contain an N-linked glycosylation site, and the 99th amino acid may contain an O-linked glycosylation site. Similarly, the 8th and 107th amino acids of EbSWP2 likely contain N-linked glycosylation sites, and the 163rd amino acid may contain an O-linked glycosylation site. The third and 133rd amino acids of EbSWP3 may contain N-linked glycosylation sites. None of the three proteins have transmembrane domains and signal peptides.

**Figure 1:**
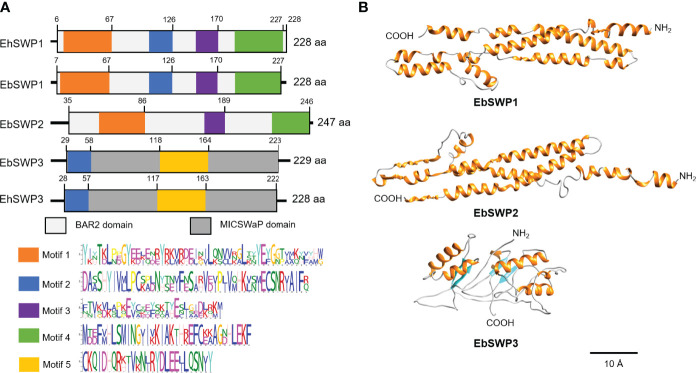
Sequence characteristics of EbSWP. **(A)** Domains and motifs in four microspore wall proteins. EhSWP1, EbSWP1 and EbSWP2 have the same BAR2 domain, while EbSWP3 has a MICSWaP domain. Altogether, five motifs were identified using MEME Suite. EbSWP1 contains the same motifs as EhSWP1, with some difference from motifs in EbSWP2, and significant difference from the motif in EbSWP3. **(B)** Structures of EbSWPs predicted by I-Tasser. EbSWP1 has 15 α-helices separated by coils (C-score = -1.13 in the range of -5 to 2) while EbSWP2 has 9 α-helices (C-score = -1.75). None of them have β-stands. In contrast, EbSWP3 contains 6 α-helix and 4 β-strands (C-score = -3.97). The α-helices are colored in orange, the β-stands are in cyan, and the coil are in gray. The N terminus and the C terminus are also labeled (Bar = 10 Å).

The motif analysis showed that the amino acid sequence of EbSWP1 and EhSWP1 share the same structure. They are similar to the structure of EbSWP2 but very divergent from that of EbSWP3 (**Figure 1A**). The sequence of the former three SWPs consist of mostly the large BAR2 domain (IPR027267), while that of EbSWP3 and EhSWP3 consist of mostly a MICSWaP domain. The predicted structure of EbSWP1 is similar to EbSWP2 (**Figure 1B**). They have high similarity to the BAR domain of humans (consensus GO scores = 0.69-0.72 in the range of 0 to 1), and consist of mostly α-helixes separated by coils that are arranged as a long strip, with the N terminus and C terminus being located at both ends of the strip. Between them, EbSWP1 has 15 α-helixes while EbSWP2 has 9 α-helixes. In contrast, Ebswp3 has a structure similar to the zinc metalloproteinase in the venom of snakes (consensus GO score = 0.41), and contains 6 α-helixes and 4 β-strands that are folded multiple times, forming a more compact tertiary structure with two domains (**Figure 1B**).

### Production of Recombinant EbSWPs in *E. coli*

**Figure 2:**
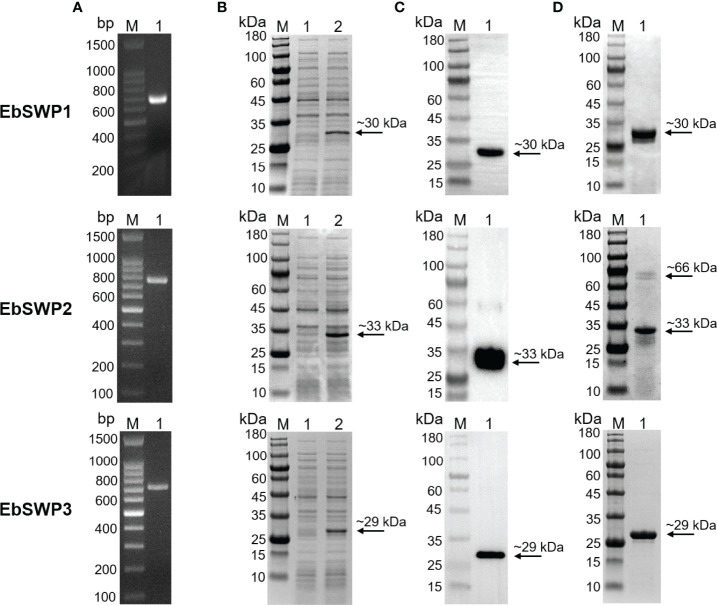
Cloning, expression, and purification of recombinant spore wall proteins EbSWP1, EbSWP2 and EbSWP3 from *Enterocytozoon bieneusi*. **(A)** PCR amplification of the target genes in genomic DNA. Lane M: molecular markers; lane 1: PCR product. **(B)** Expression of recombinant EbSWPs in *E. coli* BL21 (DE3). Lane M: protein molecular weight marker; lane 1: bacterial lysate before induction; lane 2: bacterial lysate after induction. **(C)** Western blot analysis of recombinant EbSWPs with anti-His-Tag monoclonal antibody. Lane M: protein molecular weight marker; lane 1: purified recombinant proteins from Ni-NTA affinity chromatography. **(D)** Purification of recombinant EbSWPs with Ni-NAT affinity columns. Lane M: protein molecular weight marker; lane 1: purified EbSWPs observed by SDS-PAGE.

The genes encoding the EbSWPs were cloned successfully (**Figure 2A**). All three recombinant SWPs were expressed at the predicted sizes of ~30 kDa (EbSWP1), ~33 kDa (EbSWP2) and~29 kDa (EbSWP3) (**Figure 2B**). The identity of the expressed products was confirmed by Western blot analysis, producing specific bands with the predicted sizes (**Figure 2C**). The recombinant EbSWPs were purified from lysates of transformed *E. coli* cells using the Ni-NTA resins through the His-tag incorporated into the C-terminus of the proteins. The sizes of the purified products were consistent with the expected ones in Western blot analysis (**Figure 2D**).

### Cross-Reactivity Among EbSWPs

**Figure 3:**
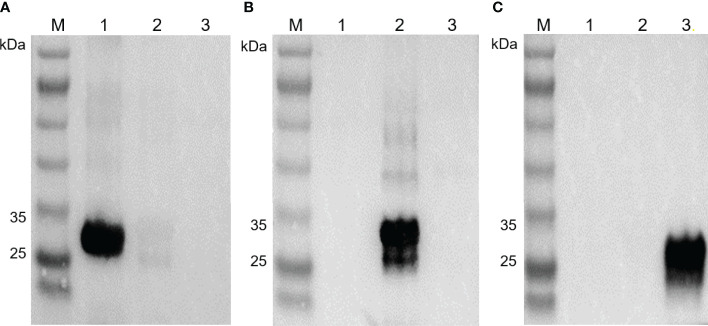
Western blot of recombinant EbSWP1, EbSWP2 and EbSWP3. **(A)** Western blot analysis of recombinant EbSWPs with anti-EbSWP1 pAb. Lane M: protein molecular weight marker; lane 1: EbSWP1; lane 2: EbSWP2; lane 3: EbSWP3. **(B)** Western blot analysis of recombinant EbSWPs with anti-EbSWP2 pAb. Lane M: protein molecular weight marker; lane 1: EbSWP1; lane 2: EbSWP2; lane 3: EbSWP3. **(C)** Western blot analysis of recombinant EbSWPs with anti-EbSWP3 pAb. Lane M. protein molecular weight marker; lane 1: EbSWP1; lane 2: EbSWP2; lane 3: EbSWP3.

The purified recombinant EbSWPs were used in the generation of pAbs. The cross reactivity of pAbs against the three EbSWPs was assessed using Western blot analysis. Anti-EbSWP1 pAb reacted strongly with recombinant EbSWP1 with no reactivities to recombinant EbSWP2 and EbSWP3 (**Figure 3A**). Similarly, EbSWP2 pAbs reacted only with recombinant EbSWP2 (**Figures 3B**), while EbSWP3 pAbs reacted only with their own target proteins (**Figures 3C**).

### Characteristics of mAb Against EbSWP1

**Figure 4:**
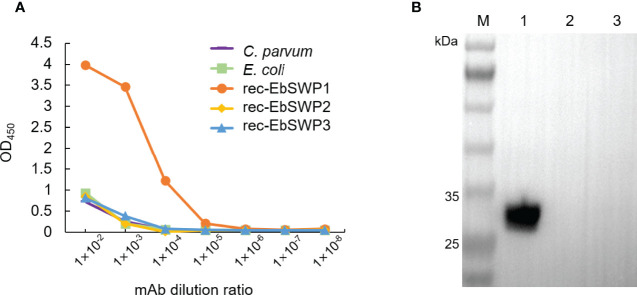
Specificity of monoclonal antibody of EbSWP1. **(A)** ELISA analysis of related antigens with anti-EbSWP1 mAb. The x-axis and y-axis are dilutions of the purified mAb and absorbance reading of the assay, respectively. **(B)** Western blot analysis of recombinant EbSWPs with anti-EbSWP1 mAb. Lane M: protein molecular weight marker; lane 1: EbSWP1; lane 2: EbSWP2; lane 3: EbSWP3.

A mAb to EbSWP1 named 2F5 was obtained after immunization of mice with recombinant protein. It belonged to IgG_1_. The specificity of mAb 2F5 was analyzed using ELISA (**Figure 4A**) and Western blot (**Figure 4B**). The mAb reacted strongly with recombinant EbSWP1 but did not react with crude antigens extracted from *C. parvum* and *E. coli* as well as recombinant EbSWP2 and EbSWP3. In the Western blot analysis of EbSWP1, the single band formed was of the expected size. The mAb did not react with recombinant EbSWP2 and EbSWP3 (**Figure 4B**).

### Detection of *E. bieneusi* Spores Using Immunofluorescent Microscopy Using mAb Against EbSWP1

**Figure 5:**
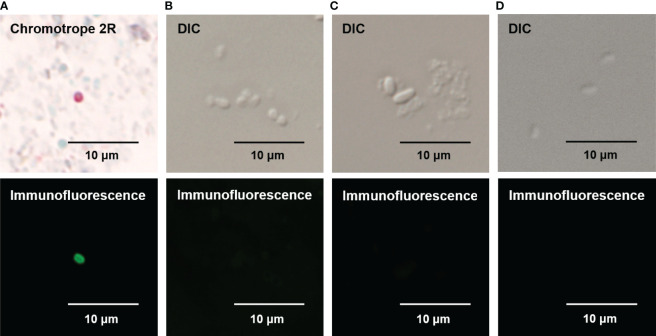
Specificity of direct immunofluorescence assay for *Enterocytozoon bieneusi* developed with mAb against EbSWP1. **(A)** Detection of *E. bieneusi* spores by Chromotrope 2R-staining (the upper panel) and immunofluorescence (the lower panel). **(B)** Detection of *E. hepatopenaei* spores using differential interference contrast (DIC) microscopy (the upper panel) and the EbSWP1-based immunofluorescence assay (the lower panel). **(C)** Detection of *E. cuniculi* spores using DIC microscopy (the upper panel) and the EbSWP1-based immunofluorescence assay (the lower panel). **(D)** Detection of *E. coli* bacteria using DIC microscopy (the upper panel) and the immunofluorescence assay (the lower panel).

FITC was conjugated to the mAb against EbSWP1. In immunofluorescent microscopy of fecal smears from *E. bieneusi*-positive samples, the FITC-labeled mAb reacted strongly with *E. bieneusi* spores. Spores were about 0.8 to 1.5 microns in size, with a wall of emerald green fluorescence (**Figure 5A**). The use of bright-field microscopy of the fecal smears stained with the chromotrope 2R-stain confirmed the identity of *E. bieneusi* spores, with the spore wall being stained bright pinkish-red (**Figure 5A**). The mAb did not react with spores of *E. hepatopenaei* and *Encephalitozoon cuniculi*, or cells of *E. coli* in the immunofluorescence analysis (**Figures 5B–D**).

Altogether, 96 fecal samples were analyzed by both the immunofluorescent microscopy using the FITC-labeled mAb and the bright-field chromotrope 2R microscopy. The former detected *E. bieneusi* spores in 63 samples, while the latter detected *E. bieneusi* spores in 58 samples. Among them, 53 samples were positive by both methods, 10 were positive only by the immunofluorescent microscopy, while 5 were positive only by the chromotrope 2R microscopy (**Table 1**). When chromotrope 2R staining was used as the gold standard, the sensitivity and specificity of direct immunofluorescence detection assay was 91.4% and 73.7%, respectively. Accordingly, the positive and negative predictive values were 84.1% and 84.8%, respectively. The positive rate of immunofluorescent microscopy (65.6%) was not significantly different from that of chromotrope 2R microscopy (60.4%) (*P* = 0.302).

**Table 1:** Performance of direct immunofluorescence assay for the detection of *Enterocytozoon bieneusi* in comparison with chromotrope 2R-based microscopy*.

Direct immunofluorescence microscopy	Chromotrope 2R microscopy		Total
	Positive	Negative	
Positive	53	10	63
Negative	5	28	33
Total	58	38	96

*Sensitivity = 53/58×100% = 91.4%; Specificity = 28/38×100% = 73.7%; Positive predictive value = 53/63×100% = 84.1%; Negative predictive value = 28/33×100% = 84.8%.

## Discussion

The SWPs of *E. bieneusi* have been characterized for the first time in the study. Using a combination of blastp and text search, we identified three *E. bieneusi* SWPs and expressed them in *E. coli*. Despite the shared structure among some of them, a good specificity of the EbSWPs was demonstrated using the pAbs generated against them. A mAb was produced against one of them (EbSWP1) and was shown to react with *E. bieneusi* of spores in fecal samples with good specificity.

*Enterocytozoon bieneusi* apparently has diverse SWPs. The EbSWP1, EbSWP2 and EbSWP3 identified in the study represent divergent SWP families. Thus far, most microsporidian SWPs identified are from *Encephalitozoon* spp. (Bohne et al., 2000; Brosson et al., 2005; Peuvel-Fanget et al., 2006; Ghosh et al., 2011), which infect vertebrates, and *N. bombycis* (Li et al., 2009; Wu et al., 2010; Cai et al., 2011; Li et al., 2012), which infects silkworms. Among the three *E. bieneusi* SWPs we identified in this study, EbSWP1 and EbSWP2 contain a BAR2 domain, therefore belong to the same SWP family (Li et al., 2009). The BAR2 domain is a dimerization motif involved in sensing and membrane curvature formation (Habermann, 2004). In contrast, EbSWP3 appears to be more divergent from other SWPs, and consists of mostly a MICSWaP domain (pfam17018**)**, which is the functional domain of another family of microsporidian spore-wall proteins (Nakjang et al., 2013). It has also been identified in divergent microsporidia, including *Encephalitozoon* spp., *Nematocida* spp., and *Hamiltosporidium* spp. (Blum et al., 2021).

Although they all belong to the SWP family of structural proteins, antibodies against EbSWP1, EbSWP2 and EbSWP3 have no apparent cross-reactivity, making them good targets for the development of specific diagnostic assays. In the present study, the pAbs against the three recombinant proteins recognized only the respective protein. For the development of an immunofluorescence assay, mAb was generated against EbSWP1. We selected EbSWP1 for the production of a mAb because its homolog in *E. hepatopenaei*, EhSWP1, was previously shown to be expressed in both the endospore and exospore of spores (Jaroenlak et al., 2018). As expected, the mAb had not cross-reactivity with recombinant EbSWP2 and EbSWP3. Therefore, it was used in the development of a direct immunofluorescence assay for the detection of *E. bieneusi* spores.

The immunofluorescence assay developed with the mAb appear to have good sensitivity and specificity. Although *E. bieneusi* is genetically related to *E. hepatopenaei*, the mAb did not react with *E. hepatopenaei* spores in immunofluorescence assay; the fluorescence-labeled mAb recognized specifically *E. bieneusi* spores. This is expected, as the amino acid sequence identity between EbSWP1 and EhSWP1 is 61% (**Figure S1**). In side-by-side comparison with chromotrope 2R staining-based microscopy, the immunofluorescence assay has slightly higher sensitivity. At present, chromotrope 2R staining-based microscopy is the most used method for microscopic detection of *E. bieneusi* in feces (Weber et al., 1992; Franzen and Muller, 2001). This method, however requires an experienced microscopist. The green immunofluorescence against a black background offered by the immunofluorescence assay, in contrast, makes the reading of stained slides easier. In previous studies, researchers used spores purified from feces as antigens to establish indirect immunofluorescence assay (Accoceberry et al., 1999; Alfa Cisse et al., 2002; Sheoran et al., 2005; Zhang et al., 2005). We have for the first time used a recombinant protein in the generation of mAbs against *E. bieneusi* spores and the development of a direct immunofluorescent assay. Using chromotrope 2R staining-based microscopy as gold standard, the sensitivity and specificity of the direct immunofluorescence for the detection of *E. bieneusi* were 91.4 and 73.7%. This is similar to the result of a direct comparison between immunofluorescence microscopy and modified trichome staining (Al-Mekhlafi et al., 2011). The low specificity of the immunofluorescence assays could have resulted from the false negative result generated by chromotrope 2R and trichome microscopy. Recently, immunofluorescence assays based on the mAbs have been marketed for the detection of *E. bieneusi* spores in fecal samples (Al-Mekhlafi et al., 2011; Jenkins et al., 2019). Compared with modified trichome staining and PCR, one kit evaluated has >95% sensitivity and specificity in the detection of *E. bieneusi* (Ghoshal et al., 2016).In conclusion, we have identified and characterized three SWPs of *E. bieneusi* for the first time. The data generated have shown that despite the shared domains among some of them, antibodies against these EbSWPs have no cross-reactivity against each other. The mAb generated against EbSWP1 was used effectively in the generation of a direct immunofluorescence assay for the identification of *E. bieneusi* in stool samples. The data and the new tool could facilitate future studies on the biology of this important zoonotic pathogen.

## Data Availability Statement

The original contributions presented in the study are included in the article/**Supplementary Material**. Further inquiries can be directed to the corresponding authors.

## Ethics Statement

The animal study was reviewed and approved by the ethics committee of the South China Agricultural University.

## Author Contributions

LX and FY conceived and designed the study. XM, HY, ZS and LS performed the experiments. XM, YG, and NL analyzed the data. XM, LX, and YF wrote the manuscript with contributions from other authors. All authors approved the final version of the manuscript.

## Funding

This work was supported by the National Natural Science Foundation of China (No. U21A20258 and 32030109), Guangdong Major Project of Basic and Applied Basic Research (No. 2020B0301030007), and Innovation Team Project of Guangdong University (No. 2019KCKTD001).

## Conflict of Interest

The authors declare that the research was conducted in the absence of any commercial or financial relationships that could be construed as a potential conflict of interest.

## Publisher’s Note

All claims expressed in this article are solely those of the authors and do not necessarily represent those of their affiliated organizations, or those of the publisher, the editors and the reviewers. Any product that may be evaluated in this article, or claim that may be made by its manufacturer, is not guaranteed or endorsed by the publisher.
