# Supplementary material for: Identification and Characterization of Three Spore Wall Proteins of Enterocytozoon Bieneusi
Source: Front Cell Infect Microbiol. 2022 Jun 20;12:808986. doi: 10.3389/fcimb.2022.808986 (PMC9251001; doi:10.3389/fcimb.2022.808986)
Supplement: Supplementary file 1 [file DataSheet_1.docx]

....|....| ....|....| ....|....| ....|....| ....|....| ....|....|

10 20 30 40 50 60

**EBI_25820_EbSWP2** METVTNNNIS KSKILSQKIN TRILKNLLSK KFKKIEYKNT DLNPGYKELE DEYKLVKHDI

**EHP00_350_EhSWP2** ..AP--KK.. TT..RETLK. .---QKKIAQ ...T.D.V.. .....FL... E.F.NIRNTA

**EBI_25395_EbSWP1** ---------- ------MYQE .---.RYIER .L.....IY. K.PD..EDI. IR.RK.RDE.

**EHP00_686_EhSWP1** ---------- ------MLED A---.RYVER .I.....IH. K.PE..E.KQ NR.RKMRDEL

**EBI_25393_EbSWP3** ---------- ---------- --------MF N.TL.AQAYG FIKYDDMSVS NNG.ETVFNV

**EHP00_695_EhSWP3** ---------- ---------- ------M.FV NYFMHAQTR- --TTHEASAT PQEQKTIFT.

....|....| ....|....| ....|....| ....|....| ....|....| ....|....|

70 80 90 100 110 120

**EBI_25820_EbSWP2** KILKNCLRAL KTYEYGNPAA QYLYMGLSWL ERKINADIVK HKGLYTNISI AGSKISKYTH

**EHP00_350_EhSWP2** .M..D..LTF .N....HSIL KNV.N.FE.V .K.L.TE..S K.E..GSLAE ..TN.A.F..

**EBI_25395_EbSWP1** G..Q.VVNG. TY....GTVM KNVAHWANIV GEST.INAI. REDI...T.T V.MQLAHTVS

**EHP00_686_EhSWP1** NV.QSVVKG. SN..F.GTIL KHFHHWSNTI SNTV.LKAL. RDDI..DTAF V.YEL.NTVG

**EBI_25393_EbSWP3** .V.APPEY.E EYQKTHESLG ID.QKIREEV .NQ..NSVRF K.NDFKIKIN LE.PQGLPIM

**EHP00_695_EhSWP3** .V.APQEYSK YYEQTHESLG AD.H.IKDEV QNEL.KSVRF RENNLKIQID LE.PA.HPVM

....|....| ....|....| ....|....| ....|....| ....|....| ....|....|

130 140 150 160 170 180

**EBI_25820_EbSWP2** DEIKKDLGIT MNNAYKSIAK SKNAFNIEVR QLIEEINKIE FQSTEINKKR KEIENIRFEL

**EHP00_350_EhSWP2** .KNR.E.A.A FQ.S.LA.SD Y.KS..S..K ...LN.DVLL SKAE..SS.. .Q.RT..YD.

**EBI_25395_EbSWP1** .KSL.EVCTE FST..EN..I E.RKM.EKME DVTD.L.NLK KKCKQ.DHQ. HIVK...YD.

**EHP00_686_EhSWP1** .KQL.EVCND FSK..EC.SE D.RKM.EKMG DIF..LSILK KKCKQ.DHQ. .TVN.L.YD.

**EBI_25393_EbSWP3** .KLNS.ICEG SITNVA.LLN EL.LADAASH YIVMLPCSPL NY.NVFESI. V..PIVQHK.

**EHP00_695_EhSWP3** .QLDETICEG SMTSVTTLLN NI.VIDASSH YIVLLPC.AD NHTEIF.SAH VDVPLVMHKV

....|....| ....|....| ....|....| ....|....| ....|....| ....|....|

190 200 210 220 230 240

**EBI_25820_EbSWP2** ENAISG--EN YDSEFIKKNR KNLS-IMCKE CMIKMNEFIK NRKIC---EI ILKFQKLHCR

**EHP00_350_EhSWP2** .M..LD--D. ..NDLV.SE. .K..-GE..Q ..SE...... DKS.G---K. .K.......K

**EBI_25395_EbSWP1** .ELLQS--NV .KEDIKNRLE .K.E-SNG.. IQEQ.TD.VH LSM.N---G. .V.IA.I.KE

**EHP00_686_EhSWP1** .EILQS--NI .KEDQKENLE .K.G-ETSEK TLVE.D..MH LSM.N---GV .K.IA.T.RE

**EBI_25393_EbSWP3** SMEC.NRVAI FQERKYDNLM ATFGNALL.I LGAPVDSYS. LNVMNTGDDG .KYSITINED

**EHP00_695_EhSWP3** NIEC.NRIAI FRESNKEMLM SSFGNAVL.A IGAPLDDYA. LTTVSNGD.G .ESHLTINEE

....|....| ....|..

250

**EBI_25820_EbSWP2** LYRNIADELD IFV----

**EHP00_350_EhSWP2** F..Q.Y.... VIEHF--

**EBI_25395_EbSWP1** FCEAAGNH.E K.N----

**EHP00_686_EhSWP1** FCKKAG.H.E K.S----

**EBI_25393_EbSWP3** AIHS.LNSRC Y.NILSL

**EHP00_695_EhSWP3** TIH..L.SKC FYNILAL

Fig S1. Alignment of amino acid sequences of SWP1, SWP2 and SWP3 of *Enterocytozoon bieneusi* and *E. hepatopenaei*. Dots denote amino acid identity to the one in the first line, while dashes dote amino acid deletions.
